# Cytological analysis of integumentary and muscular adaptations in three sand‐dwelling marine teleosts, *Ammodytes tobianus* (Ammodytidae), *Gorgasia preclara* (Congridae) and *Heteroconger hassi* (Congridae) (Teleostei; Actinopterygii)

**DOI:** 10.1111/jfb.14472

**Published:** 2020-08-18

**Authors:** Jérôme Canei, Arnaud Trupia, Denis Nonclercq

**Affiliations:** ^1^ Laboratory of Histology Biosciences Institute, Faculty of Medicine and Pharmacy, University of Mons Mons Belgium

**Keywords:** goblet cells, histoenzymology, muscle fibres, teleost, sand

## Abstract

Sandy bottoms are a ubiquitous environment found from sea bottoms to intertidal and freshwater zones. They are inhabited by many invertebrates and vertebrates which have developed morphological and physiological adaptations to sustain life under these particular conditions. Sandy habitats exhibit three potential constraints: abrasion, hypoxia and mechanical resistance. Here, three teleost species living in sandy environments were investigated: *Ammodytes tobianus* (Ammodytidae), *Gorgasia preclara* and *Heteroconger hassi* (Congridae). These teleost fishes were studied for their integument and muscular systems, which are potentially subject to sand abrasion and hypoxia, respectively. Based on histochemistry and transmission electron microscopy, we found the complex mucus system of *G. preclara* and *H. hassi* consists of two types of goblet cells and one type of sacciform cell. The secretions of both species are made of complex polysaccharides. In contrast, the scaly integument of *A. tobianus* has only a few goblet cells and no sacciform cells. We also highlighted, by immunohistochemistry, that the epidermal cell proliferation was much higher for this latter species, potentially resulting from the high rate of sand abrasion when *A. tobianus* buries itself quickly in the substrate. For all species, the major muscle fibre type was revealed by histoenzymology and corresponds to fast glycolytic fibres followed by intermediate fibres with slow fibres in the lowest proportion. *Ammodytes tobianus* possesses the highest fast fibre proportion (about 87% for *A. tobianus* and 75–78% for both garden eels). Our results provide new insights into the previously poorly studied teleost species, such as *G. preclara*, and allow us to highlight the complex skin histology of both garden eel species. Furthermore, the previously unknown muscle typing of these three species was determined.

## 1 INTRODUCTION

Sand is defined as a granular material, finer than gravels and coarser than silt, which is composed of particles between 0.05 and 2 mm in diameter. Sandy substrates are present in many terrestrial and aquatic biotopes covering a large part of our planet. For example, 20% of deserts are made of sand (Seely, 1991). In places free of ice, sandy shores (sand or a mixture of sand and rocks) represent 75% of the world shores (Brown, 2001). Psammophilous species have developed morphological, physiological and behavioural adaptations to such habitats (Etheridge, 2000). The number of species adapted to sandy substrates is high and includes many vertebrates, such as reptiles (Baumgartner *et al*., 2008; Carothers, 1986; Lamb & Bauer, 2006), amphibians (Thompson *et al*., 2005) and even some mammals (Reichman & Smith, 1990). Among them, numerous species have developed adaptations to cope with the constraints encountered in sandy substrates, like abrasion, hypoxia and mechanical resistance. The granularity of the substrate could greatly influence benthic species by, for example, leading to potential physiological adaptations to maintain an oxygenated water flow (Robards *et al*., 2000). Sand particles can also be a source of abrasion for the integument of these benthic species, and many species have developed adaptations to cope with the mechanical properties of sand particles (Wainwright & Lauder, 2017). The friction made by these particles acts as a significant selective pressure, leading to specific morpho‐physiological adaptations, such as special integument features (scales, mucus production; see Fishelson, 1996). Some species of Pleuronectiformes use thin sand layers as camouflage. Consequently, their integument has special characteristics that could retain sand particles, particularly at the dorsal epidermal level (Spinner *et al*., 2016).

Another constraint is the oxygen availability beneath sandy bottoms. The oxygen level is lower in intertidal environments subject to tides than in inferior bathymetric zones, which are constantly replenished by oxygenated currents. Among the adaptations to cope with low oxygen levels, metazoans, such as the worm *Arenicola marina*, have developed more efficient haemoglobin with a high performing affinity to oxygen (Everaarts & Weber, 1974). The choice of a suitable sandy territory, regularly washed by oxygenated water, also constitutes a behavioural adaptation to deal with potential hypoxia periods, like for the sandeel species *Ammodytes tobianus* (Robards *et al*., 2000). Finally, another constraint found in these sandy habitats is reduced locomotive ability resulting from the mechanical resistance of the substrate. Moreover, morphological specialisations are found in benthic species, as in *Heteroconger hassi*, which possesses a rigid tail with a reduced musculature, allowing this species to use its tail as a tool to dig into the sand (De Schepper *et al*., 2007). Another species, *Pisodonophis boro*, belonging to the same Anguiliformes, possesses a rigid tail but also a reinforced skull with reduced eyes, allowing this species to penetrate the substrate with its head or tail. A cinematic study performed on these two species highlighted that their locomotion is made by wavy movements different from the ones found in pelagic species (Herrel *et al*., 2011).

The first species used in the present study is the sandeel *A. tobianus* (lesser sandeel) belonging to the Ammodytidae (Uranoscopiformes), (Betancur‐R *et al*., 2017). This species lives in the North Sea, the European Atlantic coasts, and the Baltic and Mediterranean seas (O'Connell & Fives, 1995). This benthopelagic species, contrary to the garden eels that live in sandy oceanic bottoms, is found near sandy shores along intertidal zones. *A. tobianus* is very picky in choosing its habitat, preferring a sandy substrate regularly rinsed by interstitial water (Robards *et al*., 2000). The two other species we used belong to the Congridae family (Order of Anguiliformes) and more precisely to the subfamily of Heterocongrinae (Böhlke, 1957): *H. hassi* (spotted garden eel) and *Gorgasia preclara* (splendid garden eel). The spotted garden eel lives in the warm waters of the Indo‐Pacific region, and may be found in tropical regions (Miller *et al*., 2011). The splendid garden eel shares the same geographical distribution and lifestyle (Miller *et al*., 2011). The literature is scarce for both garden eel species.

The aim of our study was to investigate the morphology and physiology of two major organ systems of these teleost fishes, the integument and the muscular system. The first is related to the potential abrasion caused by the constant friction of sand particles; the second is related to its locomotion capacities and indirectly to the potential adaptations to low oxygen levels inside the sand.

## 2 MATERIALS AND METHODS

### 2.1 Animals

**FIGURE 1:**
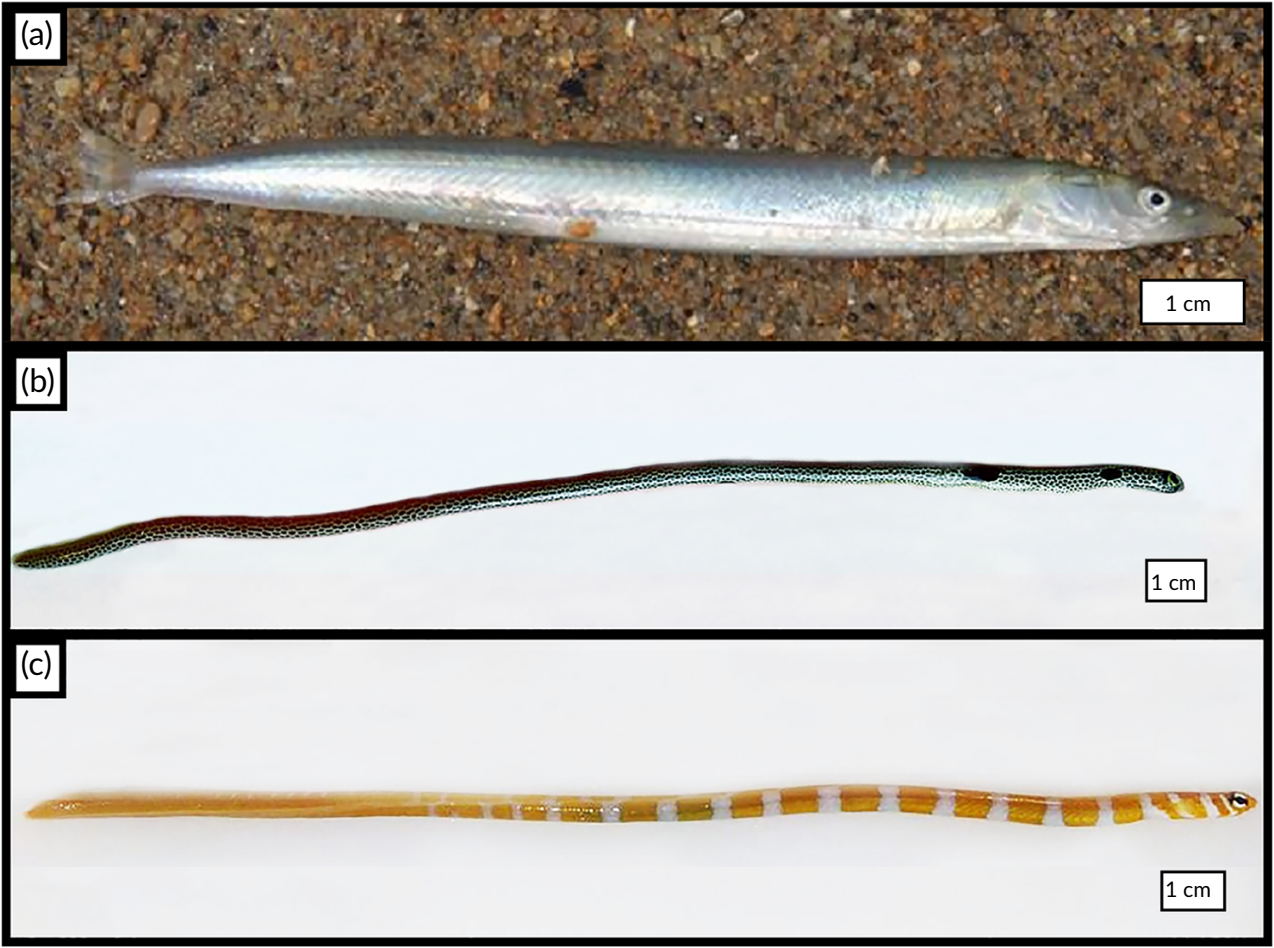
Lateral anatomical view of (a) *A. tobianus*, (b) *H. hassi* and (c) *G. preclara*

Eight adult unsexed specimens of *A. tobianus* (Figure 1a) (total length, *L*τ: 11.5 ± 0.5 cm) were collected by hand at the lowest tide of June 2018 on the beach of La Salinette in St Briac (Brittany, France). Eight unsexed individuals (total length, *L*τ: 22.03 ± 2.97 cm) of *H. hassi* (Figure 1b) were used in the experiments. These specimens were obtained from De Jong Marinelife (Spijk, the Netherlands). All specimens of this garden eel species were captured along Indonesian coasts. The fishes were located in their sandy tube when they were caught. They were then sent from Indonesia to the Netherlands at the import De Jong facilities. Eight other unsexed adult individuals (mean total length, STL: 19.33 ± 1.53 cm) of *Gorgasia preclara* (Figure 1c), from the same geographical area, were obtained from the same Dutch company. Specimens of both garden eel species were sent from the Netherlands to our laboratory by UPS and the fishes were carefully placed in bags full of sea water and air rich in oxygen for transport. All the animals were euthanized by a lethal dose (250 mg l^−1^ of water) of MS222 (tricaine methanesulfonate). The water used for the fishes was buffered by sodium bicarbonate (500 mg l^−1^). Each animal was considered euthanized when it lost the ability to swim in the aquarium. The animals were treated according to the guidelines specified by the Belgian Ministry of Trade and Agriculture and under the control of the UMONS Ethical Committee for the Survey of Experimental Studies and Animal Welfare (agreement LA1500021).

### 2.2 Histology

After euthanasia, four individuals of each species were sectioned transversally into 1 cm thick slices that were immediately fixed by immersion in Duboscq–Brazil fluid (composition: formalin/acetic acid/ethanol containing 1% picric acid/distilled water, respective proportions: 260/70/425/245 by volume) for 48 h and decalcified in 5% trichloroacetic acid for 48 h. The samples were cut into serial sections of 5 μm thicknesses on a Leica RM 2145 microtome and placed on silane‐coated glass slides after dehydration and paraffin‐embedding. After dewaxing and rehydration, the sections were stained with different staining procedures: (a) Masson's Trichrome staining (haematoxylin, Orange G, Fast Green), (b) PAS staining (periodic acid‐Schiff reaction, haemalum, Luxol fast blue), (c) Heidenhain's blue staining (haematoxylin, Ponceau Fuchsin, Aniline Blue) and finally (d) Mallory's Trichrome staining (Blue Aniline, Orange G, Ponceau Fuchsin) to allow histological examination. In addition to the classical stainings mentioned above, a more specific staining was performed to discriminate the different subpopulations of goblet cells. Alcian Blue staining (Alcian Blue, Mayer's Haemalum) allows the nature of the polysaccharides present in the mucus to be revealed. At pH 1, only the sulphated polysaccharides are stained, and at pH 2.5 both sulphated and carboxylated polysaccharides are. Tissue sections were observed on a research optical microscope (Leitz® Orthoplan) equipped with a high sensitivity camera (DFC 7000 T) and photographs were recorded using specialized software (Leica Application Suite X, LAS X; Leica company, Wetlzar, Germany). To highlight the potential lipidic secretions of glandular cells, we performed a staining test with Oild RedO on frozen fixed tissue, which allows lipids to be specifically highlighted. One body section of one individual per species was used.

### 2.3 Immunohistochemistry

#### 2.3.1 Cell proliferation

Epidermal proliferating cells were detected with a monoclonal antibody raised against proliferating cell nuclear antigen (PCNA), the accessory protein associated with DNA‐polymerase δ. This method has been detailed in a previous publication (Piron *et al*., 1998). Briefly, serial sections of the paraffin embedded body of *H. hassi*, *Gorgasia preclara* and *A. tobianus* individuals were used to study the proliferation of epidermal cells. Each centimetre along the fish's body, a 5 μm thick paraffin section was used for the immunohistochemistry. The unmasking of antigenic sites was performed by immerging the tissue sections in a Pyrex container filled with distilled water. This container was placed in a pressure cooker filled with 800 ml of distilled water at the bottom. Once firmly closed, the sections were submitted to high pressure at 120°C. After 20 min, the pressure cooker was turned off and the sections were cooled at room temperature for 30 min. After rinsing with distilled water, endogenous peroxidase activity was quenched by a 5 min exposure to 0.5% H_2_O_2_. Before applying the primary antibodies, the pretreated sections were rinsed in phosphate buffered saline (PBS) (0.04 M Na_2_HPO_4_.12H_2_O, 0.01 M KH_2_PO_4_ and 0.12 M NaCl, pH 7.4) and incubated in 0.5% casein (diluted in PBS) for 15 min. The primary antibody [mouse monoclonal (PC10) anti‐PCNA from Abcam®] was used at optimal working dilution (1:50). This antibody targets the PCNA protein (also called the DNA‐polymerase δ accessory protein) which plays an important role in DNA replication. After rinsing in PBS, the slices were treated with the complex ImmPRESS™ HRP/Anti‐MouseIgG/Polymer (Vector®, Burlingame, CA, USA) for 30 min at room temperature. Bound peroxidase activity was visualized by precipitation of 3,3′ diaminobenzidine (DAB) 0.02% in PBS containing 0.01% H_2_O_2_. Preparations were counterstained with hemalun and luxol fast blue, dehydrated and mounted with a permanent medium. Controls for the specificity of immunolabelling included the omission of the primary antibody or the substitution of nonimmune mouse serum for the primary antibody. In both cases, these controls were negative.

#### 2.3.2 Histoenzymology

Four euthanized individuals per species were used for this study. The body of each individual was transversally cut into 1 cm thick segments. All the pieces were put into a little basket filled with optimal cutting temperature glue which was itself put into an isopentane beaker. The isopentane was precooled with liquid nitrogen. All the frozen fixed tissues were stored at −80°C. The samples were cut with a thickness of 12 μm at −20°C using a CM1950 cryostat (Leica) and placed on silane‐coated glass slides. The frozen sections were then stored at −20°C until their use. The slices were treated according to the following protocol, adapted from Zghikh *et al*. (2014). Briefly, after an incubation at 37°C (pH 7.6) for 40 min in a solution of succinate 100 mM, nitroblue‐tetrazolium 1.25 mM and Meldolablau blue 8.3 mM, the slices were rinsed in physiological saline solution (0.9% NaCl). Tissue sections were pre‐incubated for 10 min at pH 10.4 and rinsed in distilled water. Thereafter, histological sections were incubated for 1 h at 37°C in a solution of adenosine triphosphate adjusted to pH 9.4. Finally, the sections were rinsed three times in 1% CaCl_2_, then in 2% CoCl_2_ for 3 min and rinsed again three times in distilled water. Revelation was performed by incubation in 1% (NH_4_)_2_S for 3 min. After rinsing for 20 min in distilled water, the preparations were dehydrated and mounted with a permanent medium.

### 2.4 Morphometric analysis

#### 2.4.1 Thickness of the integument

For the thickness of the epidermis, observations were performed on an optical microscope equipped with a high sensitivity camera and images were recorded using specialized software (all the instruments are the same as those used for general histology). We used the photographs of the Alcian Blue staining (pH 1). The measurements were performed in the dorsal, ventral, left and right regions of each body section, every 1 cm, all along the body (from head to tail) of four specimens per species. All thickness measurements were made using the public software ImageJ (Image Processing and Analysis in Java, Java 1.6.0).

#### 2.4.2 Measure of cell proliferation

For each fish, the number of epidermal proliferating cells was visually counted on each slide (four measures per section). The measurements (cell quantification) were taken on photographs with the same width corresponding to an epidermal length of 440 μm. These measurements were performed on transverse sections 1 cm apart along the animal to equally cover the body from head to tail. The sections were also divided into anterior and posterior parts, which respectively correspond to the first third and two‐thirds of the body length along the head–caudal fin axis.

#### 2.4.3 Fibre muscle typing

For each species, the number of fibres and the area occupied by each type of muscle fibre were quantified by morphometric analysis at low magnification (objective 1.5× for *A. tobianus* and objective 2.5× for *G. preclara* and *H. hassi*). The procedure was based on a hardware system consisting of a Zeiss Axioplan microscope equipped with a ProgRes C10 plus colour camera (Jenoptik, Germany) connected to an IBM‐compatible PC, and software designed for morphometry and colour analysis (KS 400 Imaging system, Carl Zeiss Vision GmbH, München, Germany). This image‐analysing system discriminated immunoreactive muscle fibres based on differences in colour and contrast. For each image (2080 × 1542 pixels), standardized microscopic fields (5,400,000 μm^2^ by image for *G. preclara* and *H. hassi*, and 21,600,000 μm^2^ for *A. tobianus*) were taken in order to cover the whole surface occupied by the musculature on the body transverse sections for the three species. For each fish (*n* = 4 per species), one transverse section was taken every centimetre along the body length (from behind the gills to the end of tail) and scanned at low magnification for morphometric analysis, as detailed above. For each species, the surfaces occupied by the three categories of muscular fibres were evaluated. These data were used to calculate the relative percentage of the musculature occupied by slow, intermediate and fast fibres.

#### 2.4.4 Scanning electron microscopy

Three small integument samples (<1 cm^3^) were collected along the body of each species (behind the gills and before the caudal fin). These were fixed in Duboscq‐Brazil's fluid for 24 h. The fixed samples were then dehydrated in graded concentrations of ethanol and critical‐point‐dried using CO_2_ as the transition fluid. They were then mounted on aluminium stubs and coated with gold in a Jeol JFC‐1100E sputter coater. The images were obtained and treated with a Jeol JSM‐7200F (JEOL Company: Tokyo, Japan) scanning electron microscope (SEM). The mean distance (width) between two consecutive micro‐ridges was measured using the aforementioned public software ImageJ (Image Processing and Analysis in Java, Java 1.6.0).

#### 2.4.5 Transmission electron microscopy

Six small integument samples (˂1 mm^3^) were taken from each individual of *G. preclara* and *H. hassi*. Three were dissected from anterior corporal parts (included between the posterior part of the gills and half of the body length) and the three others from posterior corporal parts (from half of the body length to the anterior part of the caudal fin). The tissue blocks were fixed overnight at 4°C in a solution (2% glutaraldehyde in 0.08 M sodium cacodylate buffer). The samples were rinsed three times for 10 min in buffer (0.08 M sodium cacodylate) and were then post fixed in a 1% osmium tetroxide solution (OsO_4_) for 1 h at room temperature. Thereafter, the samples were rinsed again three times for 10 min in cacodylate buffer. After fixation, the tissue blocks were progressively dehydrated in graded ethanol solutions and embedded in epoxy resin [10 g of vinyl cyclohexene dioxide (ERL‐4206), 6 g of diglycidyl ether of polypropylene glycol (DER736), 26 g of nonenylsuccinic anhydride and 0.4 g of dimethylaminoethanol]. The resin‐embedded samples were placed individually into moulds and stocked in an oven at 70°C for 24 h to allow resin polymerization. Ultra‐thin sections (80–90 nm) were obtained using an ultra‐microtome (Leica UCT; Leica, Wetzlar, Germany) equipped with a diamond‐knife. The sections were then placed on copper grids and contrasted with uranyl acetate and lead citrate. The samples were examined in a Philips TECNAI 10 Transmission Electron Microscope (Philips, Amsterdam, Netherlands).

#### 2.4.6 Statistical analysis

All the statistical analyses were carried out using the R software for statistical computing (version 3.2.1). In function of their distribution, the quantitative data obtained in this study were submitted to parametric (ANOVA one‐way when three or more groups were compared or Student's *t*‐test when two groups were compared) or nonparametric (Kruskal–Wallis when three groups or more were compared or Wilcoxon when two groups were compared) tests. *P* values lower than 0.05 are indicated on the graphs by *. All the boxplots show minimum, maximum, median, first quartile and third quartile values in each data subset.

## 3 RESULTS

### 3.1 Histology

**FIGURE 2:**
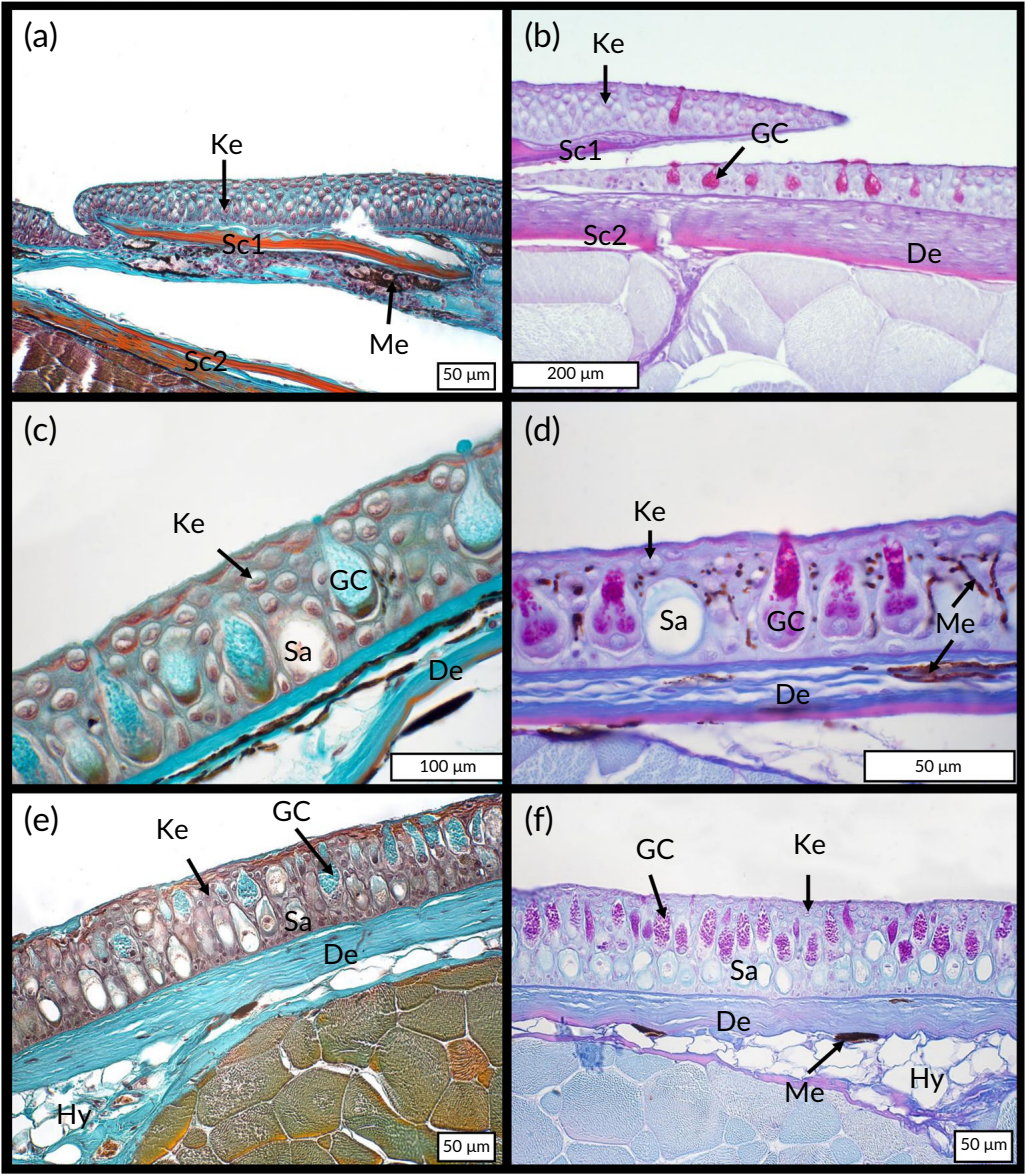
Integument morphology of *A. tobianus* [(a) and (b)], *H. hassi* [(c) and (d)], and *G. preclara* [(e) and (f)]. Paraffin embedded tissue sections were stained with Masson's Trichrome [left column, (a), (c), (e)] and with PAS‐haemalum, Luxol fast blue [right column, (b), (d), (f)]. De, dermis; GC, goblet cell; Hy, hypodermis; Ke, keratinocytes; Me, melanocytes; Sa, sacciform cell; Sc1 and Sc2, scales

The histological structure of the integument exhibits many differences between the three psammophilous Actinopterygii species. For *A. tobianus*, the integument is characterized by the presence of thin cycloid scales located in the dermis (Figure 2a). The epidermal layer is thick and composed of five to eight layers of overlapped keratinocytes. We observed no epidermal melanocytes and no sacciform or club cells. Goblet cells are present all along the body and show a higher density at the anterior part of the body. These mucous cells are preferentially located in hinge regions, at the overlapping of two successive scales (Figure 2b). The external dermis is poorly cellularized and is rich in collagen fibres. This superficial dermis is almost free of melanocytes. These are more numerous in the deeply dermis under the scales (Figure 2a), but only in the dorsal parts of the body. The hypodermis is reduced or even absent (Figure 2a,b). Scales were absent in *G. preclara* and *H. hassi* (Figure 2c–f) regardless of the body part. The epidermal layer is thick and mostly composed of glandular cells and small keratinocytes overlapping on three to four strata. The two garden eel species possess a great number of goblet cells inside their epidermis. In *H. hassi*, these cells are placed side by side at the same epidermal height (Figure 2c,d). In contrast, in *G. preclara* layering was highlighted between basal sacciform cells and apical goblet cells (Figure 2e,f). The epidermis of *H. hassi* contains clusters of melanocytes possessing thin extensions penetrating the apical strata of the cutaneous epithelium (Figure 2d) where the black spots are found on the skin. *G. preclara*, which do not exhibit dark spots or dark strips on the skin's surface, lacks intra‐epidermal melanocytes (Figure 2f). In *H. hassi*, the dermis has a low thickness and shows a loose structure (Figure 2c,d). In contrast, the dermis seems thicker and is made of a denser connective tissue in *G. preclara* (Figure 2e,f). Both species of garden eel have some dispersed melanocytes in the deeply dermis. The hypodermis is less developed in both species, but we noted some white adipocyte clusters at the junction of myotomes.

**FIGURE 3:**
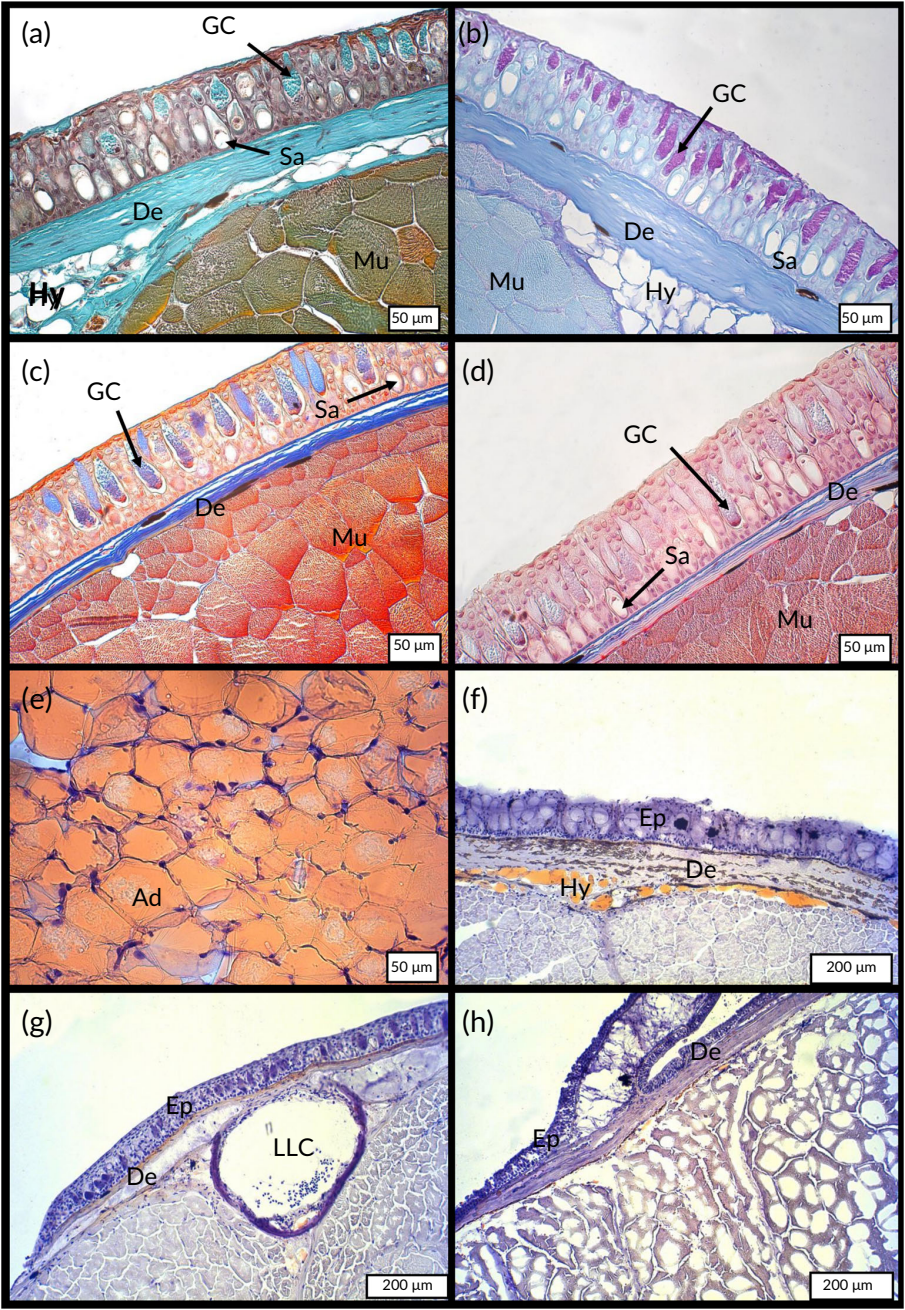
Integument morphology of *G. preclara* [(a)–(d)]. Paraffin embedded tissue sections were stained with Masson's Trichrome (a), PAS (b), Mallory's Trichrome (c) and Heindenhain staining (d). De, dermis; GC, goblet cell; Hy, hypodermis; Me, melanocytes; Mu, muscle fibres; Sa, sacciform cell. Cryosections of rat (*Rattus norvegicus)* hypoderme (e), *H. hassi* (f), *G. preclara* (g) and *A. tobianus* (h) integuments stained with Oil RedO (ORO). Ad, adipocytes; De, dermis; Ep, epidermis; Hy, hypodermis; LLC, lateral‐line canal

**FIGURE 4:**
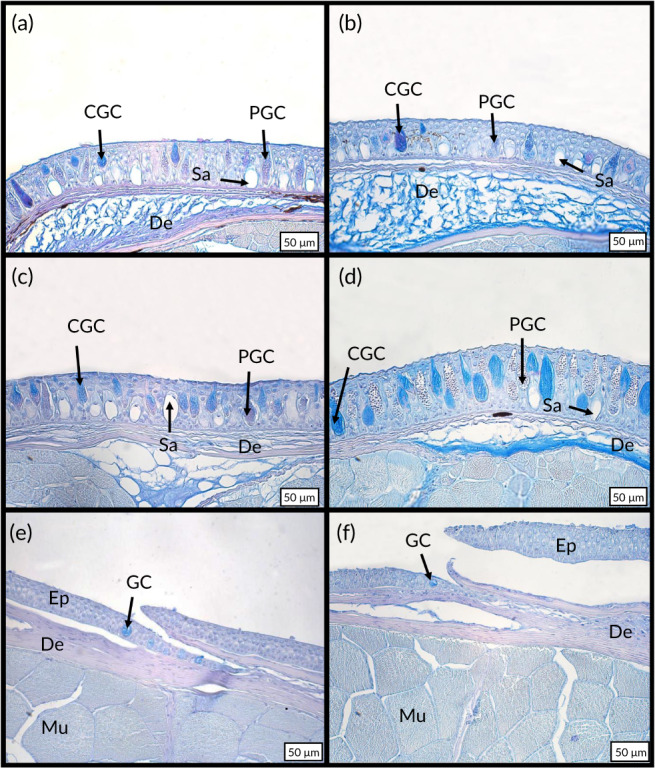
Integument morphology of *H. hassi* [(a) and (b)], *G. preclara* [(c) and (d)], and *A. tobianus* [(e) and (f)]. Paraffin embedded tissue sections were stained with Alcian Blue at pH 1 [(a), (c) and (e)] and pH 2.5 [(b), (d) an (f)]. CGC, coloured goblet cell; De, dermis; Ep, epidermis; GC, goblet cell; Mu, muscle fibres; PGC, pale stained goblet cell (granular cells); Sa, sacciform cell

The four staining methods confirmed that the epidermal goblet cells of the three studied species were stained by Masson's Trichrome (Figure 3a), PAS (Figure 3b), Mallory's Trichrome (Figure 3c) and Heidenhain staining (Figure 3d). The PAS staining indicates that the goblet cells of all three species (Figure 2b,d,f) produce a secretion composed (at least partially) of glycoproteins (mucins). In parallel, the sacciform cells were not coloured by these staining methods (Figure 3a–d). While the control composed of mammal white adipocytes was fully stained in bright reddish orange (Figure 3e), the sacciform cells (Figure 3f–h) remained unstained, exhibiting no lipidic content. In the three fish species, only a fine layer of adipocytes stained by Oild RedO were present at the hypodermis level (Figure 3f). Finally, the Alcian Blue staining method allowed us to distinguish two populations of goblet cells for both *H. hassi* (Figure 4a,b) and *G. preclara* (Figure 4c,d). One subset of goblet cells was deeply stained and the other remained very poorly contrasted, but presented a granular content. These two cell types remained stained by the PAS on consecutive sections (data not shown), pointing to a glycoprotein content in both goblet cell subpopulations. In both garden eels, the Alcian Blue staining at pH 1 (Figure 4a,c) indicated the presence of sulphated polysaccharides in a subpopulation of goblet cells which were deeply stained, and another subpopulation which were more granular and poorly stained. The same staining method at pH 2.5 (Figure 4b,d) allowed us to more precisely discriminate these two categories of goblet cells. The first presented a homogenous blue staining pattern and corresponded to the cells stained at pH 1 containing sulphated polysaccharides, whereas the second type of goblet cells presented a granular pattern and contained mucin secretions constituted of a mixture of both sulphated and carboxylated polysaccharides. In contrast, all goblet cells of *A. tobianus* were uniformly stained by Alcian Blue at both pH values (Figure 4e,f). We noted again that the sacciform cells remained unstained by Alcian Blue in both garden eels independently of the pH values (Figure 4a–d).

**FIGURE 5:**
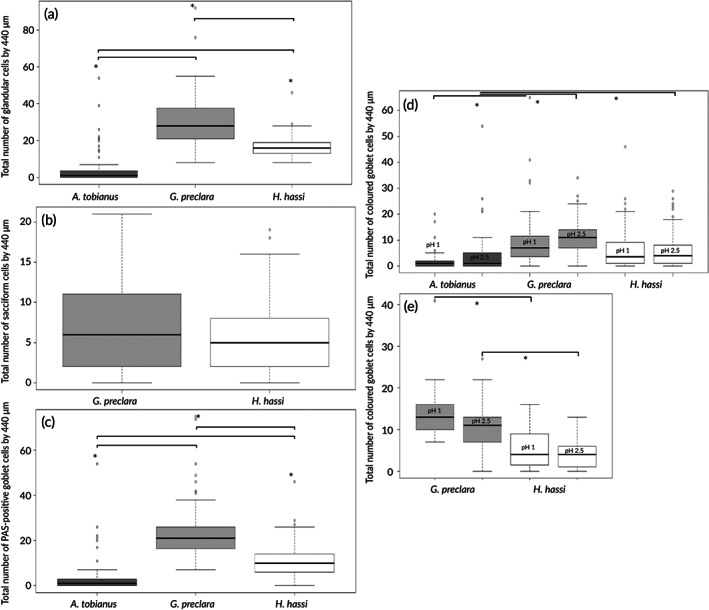
(a) Boxplots representing the total number of glandular cells for each species (*A. tobianus*, *n* = 4, *G. preclara*, *n* = 4, *H. hassi*, *n* = 4). (b) Boxplots representing the total number of sacciform cells for *H. hassi* and *G. preclara* (*G. preclara*, *n* = 4, *H. hassi*, *n* = 4). (c) Boxplots representing the total number of goblet cells (PAS‐positives cells) for each species (*A. tobianus*, *n* = 4, *G. preclara*, *n* = 4, *H. hassi*, *n* = 4). (d) Boxplots representing the total number of goblet cells deeply contrasted by Alcian Blue at pH 1 or at pH 2.5 [*A. tobianus*, *n* = 4 (pH 1), *n* = 4 (pH 2.5), *G. preclara*, *n* = 4 (pH 1), *n* = 4 (pH 2.5), *H. hassi*, *n* = 4 (pH 1), *n* = 4 (pH 2.5)]. (e) Boxplots representing the total number of goblet cells poorly contrasted by Alcian Blue at pH 1 or at pH 2.5 (corresponding to granular cells) [*G. preclara*, *n* = 4 (pH 1), *n* = 4 (pH 2.5), *H. hassi*, *n* = 4 (pH 1), *n* = 4 (pH 2.5)]. Each image had a width of 440 μm. The interspecific comparison was performed by the nonparametric Kruskal–Wallis test [or Wilcoxon for (b)] and significant differences set at *P* < 0.05. *Indicates the interspecific differences. All the boxplots show minimum, maximum, median, first quartile and third quartile values in each data subset

The morphometric analyses of the epidermal glandular cells (all categories) point to significant differences between the three species (Figure 5a). Both species of garden eels possess significantly more glandular cells with mean (±S.D.) values of 29.9 (±12.7) (per 440 μm of epidermal length) in *G. preclara* and 16.8 (±5.1) in *H. hassi*, whereas *A. tobianus* has only 3.7 (±7.6) cells. The number of sacciform cells between the two garden eel species was not statistically different (Figure 5b). *G. preclara* possessed, on average (±S.D.), 6.9 (±5.7) sacciform cells *versus* 5.8 (±4.3) in *H. hassi*. Additionally, we have shown that the number of goblet cells (all PAS‐positive cells) was statistically different between each species (Figure 5c). The mean values (±S.D.) obtained were 23 (±11.1) PAS‐positive cells/440 μm of epidermal length in *G. preclara*, 11.1 (±6.8) in *H. hassi* and only 3.0 (±6.7) goblet cells in *A. tobianus*. When we consider the results of the deeply stained goblet cell subpopulation after Alcian Blue staining at pH 1 and pH 2.5, respectively, we obtain interspecific significant differences (Figure 5d), but no intraspecific differences. Finally, the number of pale‐coloured goblet cells (corresponding to granular cells) split by the pH values was also different between the two garden eels (Figure 5e). *A. tobianus* possesses no pale granular cells stained by Alcian Blue. Beside these absolute values, we have shown that the relative percentage of each glandular cell type is quite different within each species (Table S1). Indeed, *A. tobianus* is composed entirely of goblet cells, *G. preclara* is composed of a majority of poorly stained goblet cells (granular cells) (about 41%) followed by deep coloured goblet cells (35%) and then by sacciform cells (23%). On the other hand, *H. hassi* is composed of coloured goblet cells (38%), sacciform cells (34%) and lastly poorly coloured goblet cells (granular cells) (27%).

#### 3.1.1 Epidermal thickness

**FIGURE 6:**
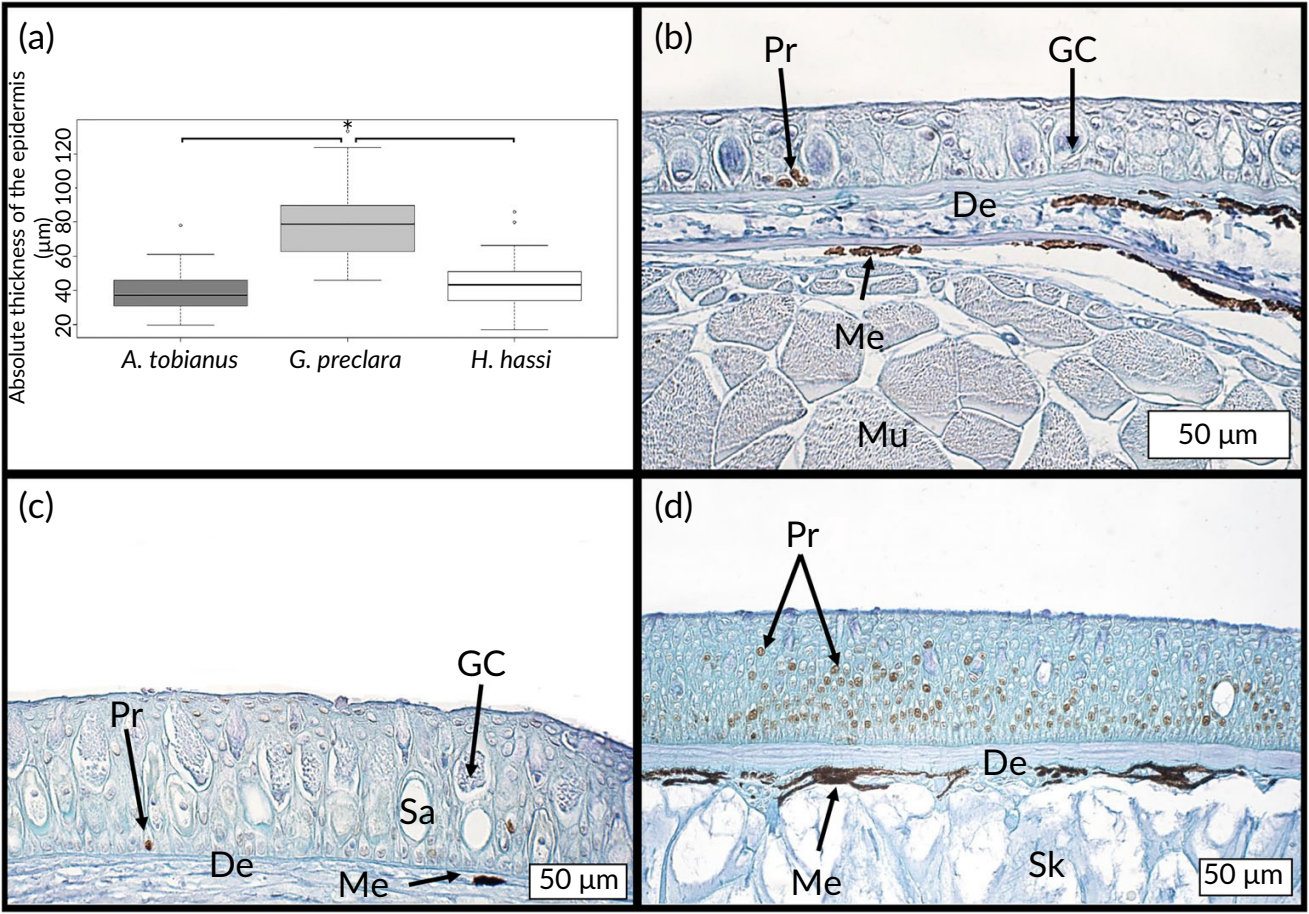
Boxplots representing the thickness values (in μm) of the epidermis of each species. The interspecific comparison was performed by the nonparametric Kruskal–Wallis test and significant differences set at *P* < 0.05 (*A. tobianus*, *n* = 4, *G. preclara*, *n* = 4, *H. hassi*, *n* = 4). All the boxplots show minimum, maximum, median, first quartile and third quartile values in each data subset (a). Microphotography of the anti‐PCNA immunolabelling performed on the integument of *H. hassi* (b), *G. preclara* (c) and *A. tobianus* (d). De, dermis; GC, goblet cell; Me, melanocytes; Mu, muscle fibres; Pr, proliferating cell targeted by anti‐PCNA antibodies; Sa, sacciform cell; Sk, skull

The thickness of the epidermis was statistically different between *G. preclara* and the two other species (Figure 6a). More precisely, *G. preclara* had a significantly thicker epidermis compared with the other species. The thicknesses of *A. tobianus* and *H. hassi* were not statistically different. The mean values (±S.D.) of thickness were 39.4 (±11.3) μm (*A. tobianus*), 77.9 (±17.9) μm (*G. preclara*) and 43.2 (±13.4) μm (*H. hassi*). No intraspecific differences were evidenced in function of corporal localization (along the head–tail axis) for all species (data not shown).

### 3.2 Immunohistochemistry

#### 3.2.1 Cell proliferation

The cells targeted by the anti‐PCNA primary antibody were clearly identifiable by the DAB precipitation (brown nuclei) (Figure 6b–d). The number of proliferating cells was very low for *H. hassi* (Figure 6b) and *G. preclara* (Figure 6c), and restricted to some scattered cells of the basal germinal stratum of epidermis. In contrast, the epidermal cell turnover was higher in *A. tobianus* where PCNA‐positive cells were not only confined to the basal germinal stratum but were also numerous in the intermediate layers of epidermis (Figure 6d).

**FIGURE 7:**
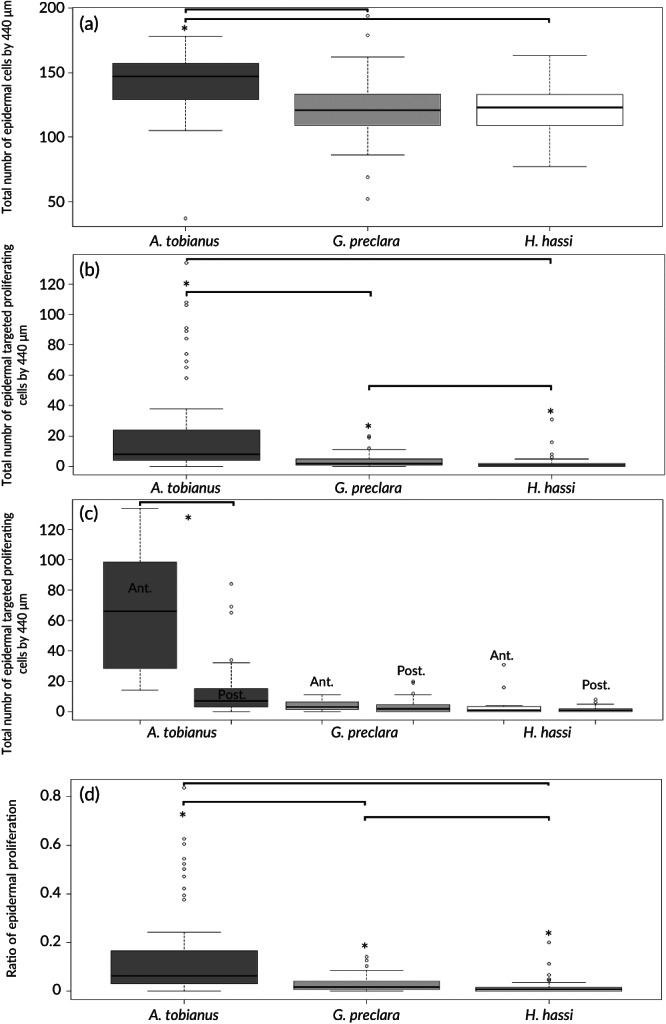
Boxplots representing for each species the total number of epidermal cells/440 μm of epidermal length (*A. tobianus*, *n* = 4, *G. preclara*, *n* = 4, *H. hassi*, *n* = 4). (a) Boxplots representing the total number of epidermal proliferating cells (PCNA‐positive cells)/440 μm of epidermal length for each species (*A. tobianus*, *n* = 4, *G. preclara*, *n* = 4, *H. hassi*, *n* = 4). (b) Boxplots representing the total number of epidermal proliferating cells evaluated separately in the anterior and posterior parts of the body [*A. tobianus*, *n* = 4 (ant.), *n* = 4 (post.), *G. preclara*, *n* = 4 (ant.), *n* = 4 (post), *H. hassi*, *n* = 4 (ant.), *n* = 4 (post.)]. (c) Boxplots representing the ratio of proliferating epidermal cells (proliferating cells/the total number of epidermal cells) (*A. tobianus*, *n* = 4, *G. preclara*, *n* = 4, *H. hassi*, *n* = 4). (d) The interspecific comparison was performed by the nonparametric Kruskal–Wallis test and significant differences set at *P* < 0.05. *Indicates the significant differences. All the boxplots show minimum, maximum, median, first quartile and third quartile values in each data subset

The statistical analyses showed us that the total number of epidermal cells/440 μm of epidermal length (Figure 7a) was significantly higher for *A. tobianus* (mean value ± S.D.: 142.56 ± 22.62) compared to *G. preclara* (mean value ± S.D.: 121.97 ± 22.03) and *H. hassi* (mean value ± S.D.: 121.53 ± 19.75). The number of proliferating cells (anti‐PCNA positive‐cells) was also significantly different between all species (Figure 7b). The mean (±S.D.) values for *G. preclara* and *H. hassi* (3.49 ± 4.12 and 1.83 ± 4.08, respectively) were close when compared to the high cell turnover recorded in *A. tobianus* (21.08 ± 29.5 proliferating cells/440 μm of epidermal length). The data split between anterior and posterior showed a significant difference between the anterior and posterior parts of *A. tobianus* (Figure 7c). The mean (±S.D.) values were 65.58 (±40.35) (anterior part) and 12.33(±16.48) cells (posterior part). No intraspecific differences were found for both garden eels. Finally, the relative percentage of epidermal cells in proliferation (corresponding to the ratio: proliferating cells/the total number of epidermal cells) was also statistically significant between *A. tobianus versus* the two species of garden eel (Figure 7d). The mean (±S.D.) values were 13.72 (±17.70) % (*A. tobianus*), 2.79 (±3.09) % (*G. preclara*) and 1.40 (±2.77) % (*H. hassi*).

#### 3.2.2 Scanning electron microscopy and transmission electron microscopy

**FIGURE 8:**
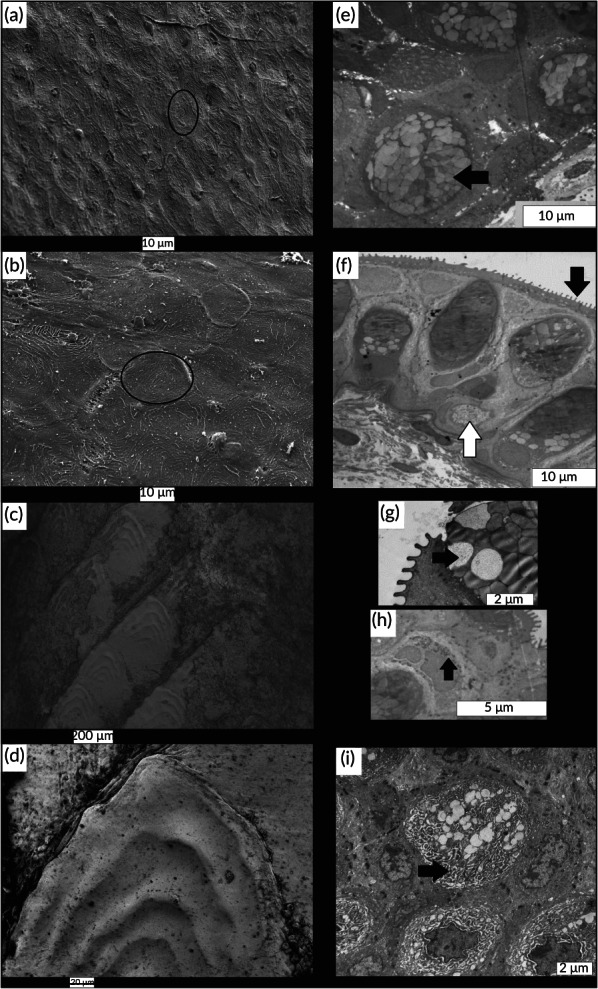
SEM of the superficial epidermis of *H. hassi* (a), *G. preclara* (b) and *A. tobianus* (c) and (d). The two first micrographs were taken at a close range to focus on the pattern of the epidermis. The black circles indicate these geometrical patterns. Micrograph (c) for *A. tobianus* was performed at a lower magnification to see the scaly pattern made by the individual cycloid scales (d). TEM of the epidermis of *H. hassi* (e) and *G. preclara* (f)–(i). Micrograph (e) illustrates typical mucous goblet cells. The black arrow indicates an individual light contrasted secretion grain. Micrograph (f) illustrates a general view of the epidermal glandular cells at low magnification. The black arrow indicates the microridges, the white one indicates a sacciform cell. Micrograph (g) presents a high magnification view of the microridges. The black arrow indicates a light grain mucous from a mucous goblet cell. Micrograph (h) shows the peculiar appearance of the second type of glandular cells exhibiting small granules of secretion (black arrow). (i) Clusters of sacciform cells exhibiting numerous dilated cisterns of rough endoplasmic reticulum (black arrow)

The SEM micrographs highlighted a thin geometrical pattern for both *H. hassi* and *G. preclara* (Figure 8a,b, respectively). These patterns looked like ‘fingerprints’ and were found at every location of the skin of both garden eel species (even on the fin rays). These structures have a diameter of about 10 μm for both species. *A. tobianus* (Figure 8c,d) presents a scaly pattern made of typical cycloid scales. These scales had a width of about 240 μm (data not shown). The transmission electron microscopy micrographs allowed us to highlight the epidermal glandular types of both garden eel species. We highlighted a first type of goblet cells, recognizable by its numerous white‐contrasted mucous secretion grains (Figure 8e–g). A second type of glandular cell was also observed, which was characterized by numerous small serous granules of secretion (Figure 8h). These granules had a round shape and exhibited a dark contrast. Sacciform cells were observed in the basal part of the epidermis (Figure 8f). These cells were characterized by a significant expansion of the rough endoplasmic reticulum (Figure 8i). The apical keratinocytes presented regular micro‐ridges clearly visible at the surface of the epidermis (Figure 8f,g).

### 3.3 Histoenzymology

#### 3.3.1 Muscle fibre typing

**FIGURE 9:**
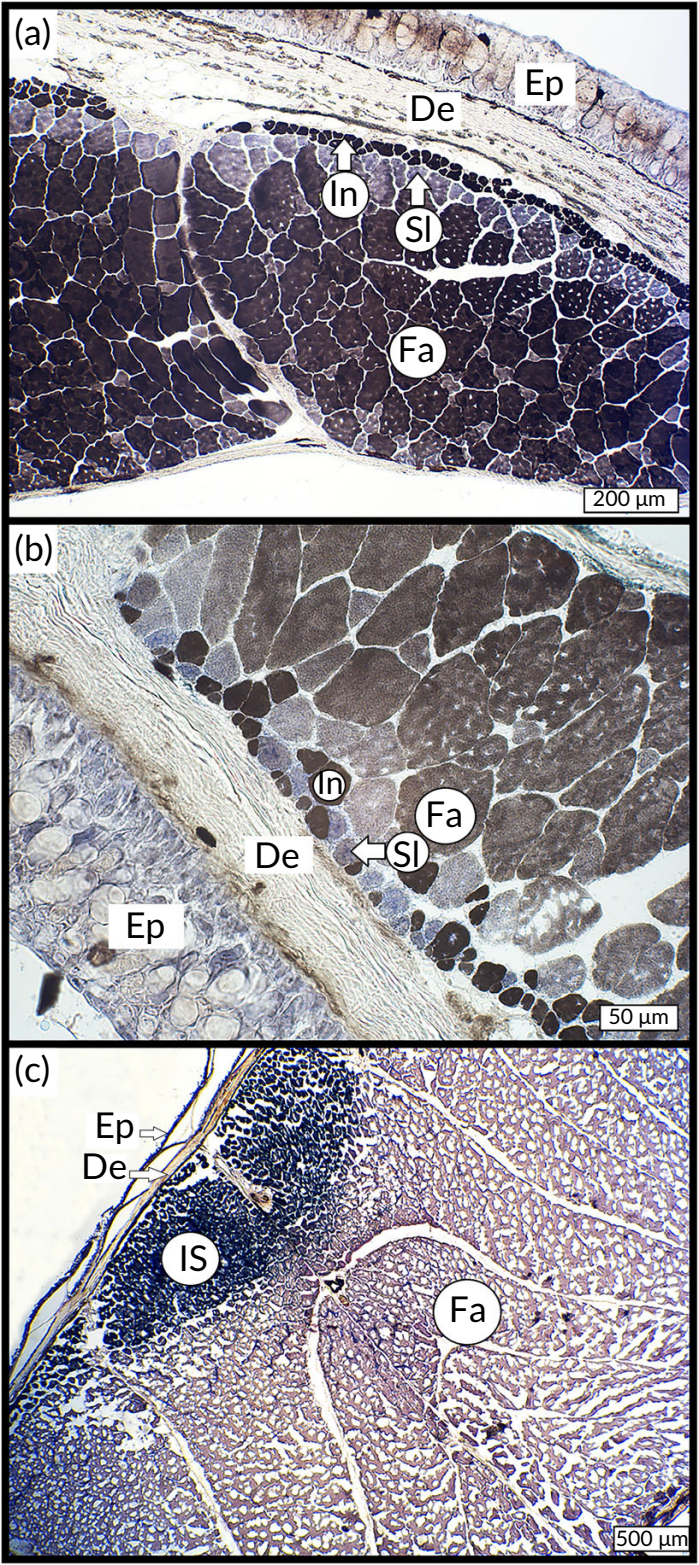
Microphotography of the histoenzymology performed on the muscle fibres of *H. hassi* (a), *G. preclara* (b) and *A. tobianus* (c). De, dermis; Ep, epidermis; Fa, fast fibres (dark brown); In, intermediate fibres (black); IS, mixed population of intermediate and slow fibres; Sl, slow fibres (blue)

**FIGURE 10:**
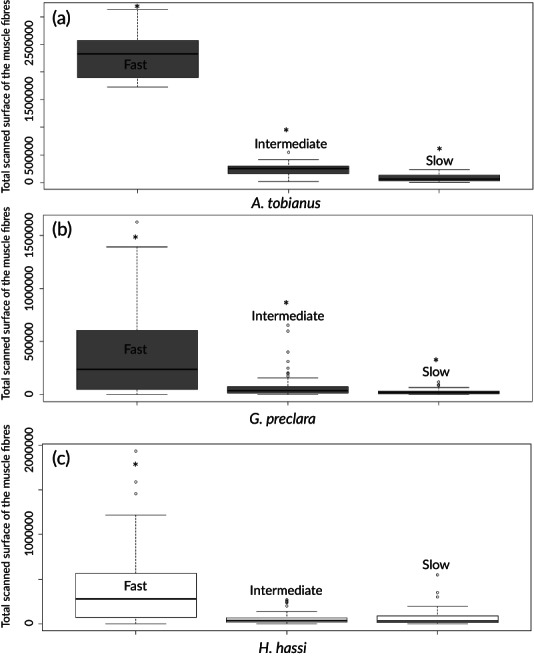
Boxplots representing the absolute surface occupied by each type of muscle fibre (fast, intermediate and slow) per microscopic field of 21.6 × 10^6^ μm^2^ for *A. tobianus* (a) (fast, *n* = 4, intermediate, *n* = 4, slow, *n* = 4), and per microscopic field of 5.4 × 10^6^ μm^2^ for *G. preclara* (b) (fast, *n* = 4, intermediate, *n* = 4, slow, *n* = 4) and *H. hassi* (c) (fast, *n* = 4, intermediate, *n* = 4, slow, *n* = 4). The assessment of intraspecific differences was performed by the nonparametric Kruskal–Wallis test and significant differences set at *P* < 0.05. *Indicates the groups that are statistically different. All the boxplots show minimum, maximum, median, first quartile and third quartile values in each data subset

The histoenzymology performed on cryosections of our three species revealed three types of muscular fibres identifiable by their colouration (Figure 9). For the three species, the fast fibres were coloured in brown, the intermediate fibres in black and the slow fibres in blue. The intense blue colouration of the slow fibres was due to the targeting of the succinate dehydrogenase located inside the mitochondria, giving a spotted pattern to these fibres. The fast‐myosin ATPase activity gives the brown colour to fast muscular cells. The combination of the brown precipitate (resulting from ATPase activity) and the blue colour (associated with succinate dehydrogenase activity) gives a dark black staining pattern to the intermediate fibres. The total scanned surface of muscles fibres analysed by morphometry gave us insights about the relative proportions of fibre types. The statistical analyses showed that the surface occupied by each fibre type per microscopic field was significantly different for *A. tobianus* (Figure 10a). Indeed, the surface occupied inside myotomes by fast fibres was significantly higher than those of slow and intermediate fibres. On the other hand, intermediate fibres occupied a larger surface of myotomes compared to slow fibres. The same intraspecific differences were found for *G. preclara* (Figure 10b) and *H. hassi* (Figure 10c). For the latter, there was, in contrast, no statistically significant difference between the slow and the intermediate fibres. As the total scanned surfaces were not comparable because of the absolute size differences of the three species (*A. tobianus* being much broader than the garden eels), we estimated the relative proportions of each fibre type between the species (Supporting Information Table S2). The majority of muscle fibres were composed of fast fibres for all species, followed either by intermediate or slow fibres.

## 4 DISCUSSION

Sandy substrates, because of their constraints, subject living organisms to significant selective pressures. The behavioural ecology of the genus *Solea* (Pleuronectiformes) is, for example, characterized by a hiding behaviour that is helped by morphological adaptations at the skin level (Spinner *et al*., 2016). In contrast, sand‐burying species must resist sand abrasion by producing protective mucin or by developing a thick integument acting as armour.

We note that the presence of melanocytes in the epidermis is uncommon among the teleost fishes, where this kind of cell is almost exclusively located in the dermis for a lot of species. All the histological staining methods performed on the tissues of both garden eel species did not allow us to confirm the precise nature of the sacciform cell secretions. These cells remained uncoloured with all the stains and thus led us to conclude that they contain no big molecules, like sulphated or carboxylated polysaccharides. This lack of colouration could be an indication that these secretions are made of small uncoloured, hydrophilic molecules (ions, amino acids, catecholamine) or even of peculiar proteins. We highlighted, through electron microscopy, the presence of numerous dilated cisternae of rough endoplasmic reticulum within these sacciform cells. This characteristic points to a high rate of protein synthesis. According to the scarce literature on the subject, many hypotheses have been suggested concerning the nature of secretions and the physiological role played by sacciform cells. The study of Mittal and Munshi (1971) performed on three fossorial teleost species proposed a hypothesis suggesting that the sacciform cells are a holocrine gland instead of a merocrine one. The cells that we have described are unicellular and do not match with the holocrine gland hypothesis detailed in their study, these theoretically requiring multicellular components. In *G. preclara* the sacciform cells are exclusively located at the basal epidermal level. In *H. hassi* they are found at mid‐height between the basal and the apical epidermis, but generally closer to the basal epidermis. These observations contradict the Mittal and Munshi (1971) hypothesis about migrating cells, at least for our two garden eel species. It was not possible to identify any lipidic content for the paraffin embedded tissues, the fixation process eliminating all the fatty components. The frozen‐fixed tissue allowed us to perform Oil RedO staining, which did not colour the sacciform cells. In light of these results, we can confirm that the sacciform cells of both garden eel species seem to be free of lipidic content (fatty acids or triglycerides). We can also exclude an albuminous secretion because of the negative results obtained with Mallory's Trichrome. Another study, by Fishelson (1996), was performed on a garden eel species, *Gorgasia sillneri*. In this study, the author offered many hypotheses on the role and nature of the sacciform cells and highlighted the PAS coloured cells. In light of our results, where no sacciform cells were coloured by this staining, we suppose that Fishelson (1996) wrongly named these cells, which were actually a subset of goblet cells. Our last hypothesis is that these sacciform cells are in fact epidermal club cells. These cells, which are common in the superorder of Ostariophysi, do not discharge their content until cutaneous injuries or stress occur (Chivers *et al*., 2007; Manek *et al*., 2013). The substances present in the secretions are made of amino acids or catecholamine that act as chemical alarm cues, or even small peptides acting as biocide. Beyond the Ostariophysi, some species such as *Salmo trutta* and *Salvelinus alpinus* exhibit epidermal sacciform cells which seem to contain proteinaceous compounds (Pickering & Fletcher, 1987). In this particular study, the proteinaceous material of those cells was specifically highlighted by stainings such as mercuric bromophenol. We must note that the presence of sacciform cells with a proteinaceous content, or the presence of epidermal club cells, in other teleost species could point towards the presumption that the so‐called sacciform cells of *H. hassi* and *G. preclara* could secrete small molecules made of amino acids or even small proteins. The lack of staining of our sacciform cells, no matter the fixation and staining method, might be an indirect indication of such compounds. Another related hypothesis could be that this cell type could produce proteins acting as a glue to clump the grains of sand from the tube in which the garden eels live and keep it open. The presence of numerous dilated cisternae of the rough endoplasmic reticulum points to a high rate of protein synthesis, which could be involved in this hypothetical function.

Concerning the goblet cells, which constitute a second cellular secretory type, these were present in all three species. The positive results obtained with PAS, Mallory's Trichrome, Masson's Trichrome and Alcian Blue give us insights about the nature of their secretion. The secretions of all three species are, at least partially, made of sulphated polysaccharides and, to a lesser extent, of carboxylated ones.

Two categories of goblet cells were identified: a typical subset which was deeply stained by the Alcian Blue and another which was poorly stained by this method. This last category of cells was found in both garden eel species but was absent in the integument of *A. tobianus*. These goblet cells, poorly contrasted by Alcian Blue, present an accumulation of fine granules in the apical part of the cytoplasm. These secretory granules were deeply coloured by the PAS, which indicates the glyco‐proteinaceous nature of the secretions. We should note that the Alcian Blue staining method gives just an indication about the polysaccharide content and only a thorough biochemical analysis would give the exact nature of the mucins secreted by these two types of goblet cells. Finally, the observations in electron microscopy highlight the presence of glandular cells with numerous small serous granules in the integument of garden eels that could correspond to this type of glandular cell. As previously mentioned for sacciform cells, it is also possible that this cell type contributes to producing proteins acting as glue to clump the grains of sand from the tube in which the garden eels live and keep it open. Both Fishelson (1996) and Mittal and Munshi (1971) observed goblet cells in the integument of garden eels, although they did not mention any differentiation inside this cellular type.

As the structure of the epidermis is different between the two garden eel species and the sandeel *A. tobianus*, we expected to observe a very different localization for the goblet cells. For *A. tobianus*, these cells were in small numbers and mainly localized in the overlapping zone of two consecutive scales. We hypothesise that the mucus secreted in these zones could ease the shift between the scales when the animal is swimming or digging in the sand. Moreover, this mucous film could prevent the accumulation of sand grains in these hinges when the animal is completely buried. The large number of glandular cells in both garden eels suggests that the mucus surrounding the body plays an important function in the resistance to abrasion. Mittal and Munshi (1971) and Fishelson (1996), proposed this hypothesis. The mucus could play a lubricant role and could reduce the mechanical injuries due to abrasion. Fishelson (1996) goes further, pointing out the role of the micro‐ridges that he observed in *G. sillneri* and that we have observed in both garden eel species. These superficial micro‐ridges could improve the resistance to abrasion by anchoring the mucus at the epidermal surface, prolonging its protective effect. These micro‐ridges were observed at every level of the body of *G. preclara* and *H. hassi*, even at the fin rays level (not shown). It is noteworthy that other roles are attributed to the mucus produced by the goblet cells. Among the main roles, which are not mutually exclusive, are protection and osmoregulation of the gills (Robards *et al*., 2000), ion and electrolyte regulation (Shephard, 1994), repulsion of predators (Fishelson, 1996) and protection against bacteria (Ebran *et al*., 2000).

The results obtained concerning the cellular epidermal proliferation and the epidermal thickness point to major interspecific differences. *A. tobianus* possesses a slightly thinner epidermis compared to *G. preclara*, but the sandeel exhibits a 10× higher epidermal cell turnover compared to both garden eels. In contrast to most of the vertebrates, where proliferating cells are restricted to the basal germinal layer of epidermis, in *A. tobianus* these cells are located on many levels, from the basal layer to the mid strata of the epidermis. Moreover, we have brought to light that this proliferation is much greater in the anterior part of the body. We could make the assumption that this is caused by the peculiar behaviour of this species, the lesser sandeel digging into the sand using its head. Considering that the mucus production is assuredly reduced for this species, the injuries caused by abrasion are inevitable (Robards *et al*., 2000). The hypothesis that seems the most probable is that *A. tobianus* copes with this mechanical constraint, with the help of a great cell turnover, particularly at the head level. We find this kind of adaptation in human beings in which the cell proliferation rate is higher in the parts of the body where there is thick skin submitted to frequent friction, such as the palms of the hands and the soles of the feet. Thus, the epidermis of the sole of the foot is entirely renewed every 20–30 days (Iizuka, 1994). In both garden eel species, the cellular epidermal turnover is much slower. The PNCA‐positive cells were rather scarce and were limited to the stratum germinativum. We hypothesise that epidermal glandular cells produce a glycoprotein film stabilizing the walls of tunnels in which they live, which would allow the friction of the epidermis to be reduced. This would explain why we observed a lesser proportion of epidermal cellular turnover for *G. gorgasia* and *H. hassi*. The epidermis of *H. hassi* is thinner that of *G. preclara*, which could be explained by the fact that the latter possesses an epidermis with more stratification. Considering the fact that both species share the same lifestyle and present the same cellular types, we have no convincing explanation concerning this interspecific difference.

In general, the musculature represents 40–60% of the body mass of teleost fishes (Alami‐Durante & Rescan, 2003). This musculature is organized in structural units called myotomes, which vary greatly according to the species, the developmental stage and even the position inside the animal (Johnston *et al*., 2011). This structure, in its most classical pattern (Altringham & Ellerby, 1999; Zhang *et al*., 1996), is found in *A. tobianus*, whereas both garden eels have a much more complex pattern. Inside these muscular bundles three fibre types are found: slow, intermediate and fast.

Numerous factors influence the distribution of muscular fibres among teleost fishes, particularly temperature and oxygen availability (Johnston, 2006), and many fishes are capable of great acclimation, which can affect the fibre typing in function of the location and the time at which a fish is captured (Sidell, 1980).

As for the epidermis, the muscular system presents major differences between our three psammophilous fishes. *A. tobianus* possesses a standard myotome structure. In this species, which lives part of the time in cold and turbulent waters rich in oxygen, we could expect a higher proportion of slow and intermediate fibres compared to both garden eel species, which live in warm waters. In contrast to this assumption, the slow fibres represent only a weak proportion of muscles fibres, about 3%. On the other hand, intermediate fibres represent about 10% of the total muscular fibres. The reasons behind these numbers are not necessarily due to the environmental oxygen level but probably due to the fossorial lifestyle of *A. tobianus*. Once buried, the sandeels capture the small amount of oxygenated water within the sandy substrate. They are still able to live there, but they capture 40–50% less oxygen than in the water column (Behrens *et al*., 2007). This weak level of available oxygen in the near substrate surrounding the animal could explain the very high proportion, more than 80%, of fast fibres, as these rely on anaerobic glycolysis and thus consume less oxygen. When discussed in the literature we see that the relative proportion of fast fibres can vary along the body from 66.3% to 82.5% for a nonpsammophilous and pelagic species, such as *Stenotomus chrysops* (Zhang *et al*., 1996). The study of Drazen *et al*. (2013) indicates that benthic fish species seem to possess on average a lower proportion of slow fibres (2.1%) when compared to benthopelagic species (7.6–10.2%). The study of Altringham and Ellerby (1999) also mentions the fact that constantly swimming pelagic species tend to have more slow fibres.

In comparison with our results, we see that both garden eels, considered as benthic species, exhibit a higher proportion of slow fibres than the benthopelagic species *A. tobianus*. The fact that we have obtained a lower proportion of slow muscles fibres for the benthopelagic and more active *A. tobianus*, in contradiction of the prementioned studies, pushes us towards a hypothesis linked to the poor level of oxygen in the environment. Indeed, despite being notably more “active” than both garden eels, this species has to face regularly poor oxygen levels when buried in the sand.

Moreover, the small amount of oxygen inside the blood circulation may be aimed towards vital organs (brain, heart, kidneys). Beside hypoxia, other factors could equally play a role in favour of a rich content of fast fibres (and therefore low intermediate and slow fibres content). Coarse sand (1–2 mm diameter) induces a mechanical resistance such that the animal has to perform powerful body movements to bury itself, and the fast fibres have the ability to furnish such power. Indeed, *A. tobianus* is able of very quick movements reaching 5 m s^−1^ (Robards *et al*., 2000). Another possible explanation could be the intense predation to which *A. tobianus* is subjected, considering that they are an essential food resource for many predatory fishes and bird species (Daunt *et al*., 2008; Robards *et al*., 2000). To survive, only powerful and fast movements, potentially provided by the higher fast fibre content, are required. *G. preclara* and *H. hassi* exhibit an uncommon myotome structure, which is separated into four distinct muscle bundles. The results seem to indicate a higher proportion of slow and intermediate fibres in both species in comparison to *A. tobianus*. This could be a handicap in view of the lifestyle of these garden eels living in warm tropical waters, which are poorer in oxygen, despite being benthic and low active species. However, some hypotheses could be proposed to explain these results. Both *G. preclara* and *H. hassi* live in tunnel nests (Tyler & Smith, 1992) open to the water column, which gives them the advantage of having constant access to the more oxygenated pelagic waters. In contrast to *A. tobianus*, these species constantly live in the same place and do not seem to be hunted by larger species. Thus, with such weak predation they would not need as many fast fibres allowing as fast movements as the sandeel. In summary, the higher relative proportion of fast fibres for *A. tobianus* could be due to potential hypoxia, mechanical resistance and escape and hunting behaviour, all of which are nonmutually exclusive.

In conclusion, the adaptations observed in the integument and muscles are very different between the sandeel *A. tobianus* and the garden eels *G. preclara* and *H. hassi*. These differences are related to the very different behaviours adopted by these two psammophilous fish families.

## AUTHOR CONTRIBUTIONS

J.C. contributed to the ideas, data analysis, data generation and manuscript preparation. A.T. contributed to the ideas, data analysis and data generation. D.N. contributed to the ideas, data analysis, manuscript preparation and funding.

## Supporting information


**Supporting Information Table S1**. The relative proportion as a percentage (mean value ± S.D.) of each glandular cell type present in epidermis of *Ammodytes tobianus*, *Gorgasia preclara* and *Heteroconger hassi*. The total for each species corresponds to 100% of glandular epidermal cells
**Supporting Information Table S2** The relative proportion as a percentage (mean value ± S.D.) of each muscle fibre type in total myotomes scanned on cross‐sections every centimetre along the body of *A. tobianus*, *Gorgasia preclara* and *Heteroconger hassi*. The sum of fast, intermediate and slow fibres corresponds to 100% of muscular fibres for each species
