# Supplementary material for: Cytological analysis of integumentary and muscular adaptations in three sand‐dwelling marine teleosts, Ammodytes tobianus (Ammodytidae), Gorgasia preclara (Congridae) and Heteroconger hassi (Congridae) (Teleostei; Actinopterygii)
Source: J Fish Biol. 2020 Aug 18;97(4):1097–112. doi: 10.1111/jfb.14472 (PMC7590194; doi:10.1111/jfb.14472)
Supplement: Supplementary file 1 — Supporting Information Table S1. The relative proportion as a percentage (mean value ± S.D.) of each glandular cell type present in epidermis of Ammodytes tobianus, Gorgasia preclara and Heteroconger hassi. The total for each species corresponds to 100% of glandular epidermal cells Supporting Information Table S2 The relative proportion as a percentage (mean value ± S.D.) of each muscle fibre type in total myotomes scanned on cross‐sections every centimetre along the body of A. tobianus, Gorgasia preclara and Heteroconger hassi. The sum of fast, intermediate and slow fibres corresponds to 100% of muscular fibres for each species [file JFB-97-1097-s001.docx]

| Species | Alcian blue deeply stained goblet cells | Alcian blue poorly stained goblet cells (granular cells) | Sacciform cells |
| --- | --- | --- | --- |
| *Ammodytes tobianus* | 100 | 0 | 0 |
| *Gorgasia preclara* | 35.5 ± 0.3 | 41.3 ± 0.2 | 23.2 ± 0.2 |
| *Heteroconger hassi* | 38.1 ± 0.5 | 27.6 ± 0.2 | 34.3 ± 0.3 |

**Table A1.** The relative proportion in % (mean values ± Standard Deviation) of each glandular cell type present in epidermis of *A. tobianus*, *G. preclara* and *H. hassi*. The total for each species corresponds to 100 % of glandular epidermal cells.

**Table A2.** The relative proportion in % (mean values ± Standard Deviation) of each muscle fibre type in total myotomes scanned on cross-sections every centimeter along the body of *A. tobianus*, *G. preclara* and *H. hassi*. The sum of fast, intermediate and slow fibres corresponds to 100 % of muscular fibres for each species.

| Species | Fast fibres | Intermediate fibres | Slow fibres |
| --- | --- | --- | --- |
| *Ammodytes tobianus* | 87.2 ± 16.8 | 9.6 ± 4.9 | 3.2 ± 2.45 |
| *Gorgasia preclara* | 78.2 ± 79.7 | 16.5 ± 26.2 | 5.3 ± 4.8 |
| *Heteroconger hassi* | 75.6 ± 80.1 | 11.8 ± 13.1 | 12.6 ± 17.6 |
